# Functional Movement Disorders during COVID-19: Psychological Distress, Affective Temperament and Emotional Dysregulation

**DOI:** 10.3390/jpm13020175

**Published:** 2023-01-19

**Authors:** Delfina Janiri, Martina Petracca, Lorenzo Moccia, Marcella Solito, Maria Rita Lo Monaco, Maria Luana Cerbarano, Carla Piano, Isabella Imbimbo, Marco Di Nicola, Alessio Simonetti, Gabriele Sani, Anna Rita Bentivoglio

**Affiliations:** 1 Department of Psychiatry, Fondazione Policlinico Universitario Agostino Gemelli IRCCS, 00168 Rome, Italy; 2 Department of Psychiatry and Neurology, Sapienza University of Rome, 00100 Rome, Italy; 3 Movement Disorders Unit, Fondazione Policlinico Universitario Agostino Gemelli IRCCS, 00168 Rome, Italy; 4 Department of Neuroscience, Università Cattolica del Sacro Cuore, 00168 Rome, Italy; 5 Medicine of Ageing, Fondazione Policlinico Universitario Agostino Gemelli IRCCS, 00168 Rome, Italy; 6 Institute of Internal Medicine and Geriatrics, Università Cattolica del Sacro Cuore, 00168 Rome, Italy; 7 Clinical Psychology Unit, Fondazione Policlinico Universitario Agostino Gemelli IRCCS, 00168 Rome, Italy

**Keywords:** functional movement disorders, COVID-19, psychological distress, stress, emotional dysregulation, temperament

## Abstract

**Background and objective**: Functional movement disorders (FMD) represent a spectrum of psychosomatic symptoms particularly sensitive to stress. The COVID-19 pandemic has increased psychological distress worldwide and may have worsened FMD. The study aimed to confirm this hypothesis and to test whether in FMD there is a relationship between affective temperament, emotional dysregulation and psychological distress due to the pandemic. **Methods**: We recruited individuals with FMD, diagnosed them according to validated criteria and matched them with healthy controls (HC). Psychological distress and temperament were obtained using the Kessler-10 and the Temperament Evaluation of Memphis, Pisa and San Diego Autoquestionnaire, respectively. We used bootstrapped mediation analysis to test the mediator role of emotional dysregulation on the effect of temperament on psychological distress. **Results**: The sample consisted of 96 individuals. During the pandemic, 31.3% of the patients reported the need for urgent neurological care, and 40.6% reported a subjective worsening neurological condition. Patients with FMD presented with more psychological distress during the COVID-19 pandemic (F = 30.15, df = 1, *p* ≤ 0.001) than HC. They also reported more emotional dysregulation (F = 15.80, df = 1, *p* ≤ 0.001) and more cyclothymic traits (F = 14.84, df = 1, *p* ≤ 0.001). Cyclothymic temperament showed an indirect effect on COVID-19-related psychological distress, mediated by deficits in emotion regulation mechanisms (Bootstrapped LLCI = 0.41, ULCI = 2.41). **Conclusion**: Our results suggest that emotional dysregulation may represent a dimension mediating cyclotimic temperament response to the stressful effect of the pandemic and provide insight for developing intervention policies.

## 1. Introduction

The coronavirus disease 2019 (COVID-19) pandemic represents a great stressor to global health [1]. Early data reported an elevated rate of psychological distress following the COVID-19 outbreak [2,3] and highlighted that individuals with neurological and psychiatric diseases could be particularly at risk [4,5,6]. Recent findings specified that COVID-19-related stress response could be influenced by individual characteristics, such as affective temperament traits and emotion regulation mechanisms [7]. Temperament is defined as a temporally stable biological core of personality, refers to individual activity level and moods, and is directly linked with emotion regulation [8].

Among neuropsychiatric conditions, functional (psychogenic) movement disorders (FMDs) represent a spectrum of neurological symptoms particularly sensitive to stress [9,10]. FMDs entail involuntary (abnormal) movements that are significantly altered by distractive maneuvers and are inconsistent and incongruent with phenomenology described in neurological diseases [11]. They can present in overlap with other neurological conditions [12] and can greatly impact the quality of life [13,14]. Up to 18% of patients with a movement disorder may be diagnosed as FMDs. Over the years, stricter clinical criteria and investigational advances have allowed the diagnosis of FMD to be more accurate [15] and confirmed psychological distress as a predisposing, precipitating and perpetuating factor for FMD [9]. Accordingly, a recent study on a large Italian cohort of 410 patients [16] highlighted that individuals with FMD frequently present with non-motor symptoms, especially anxiety, fatigue and pain.

## 2. Aims

The aims of our study are twofold: (1) to monitor COVID-19 pandemic-related psychological distress in a sample of patients with FMD, diagnosed according to strict and validated clinical criteria [6] compared to a demographically matched sample of healthy individuals (HC); (2) to determine if in FMD there is an association among temperament, emotional dysregulation and psychological distress related to the pandemic. Specifically, we hypothesized that temperament might influence psychological distress during the COVID-19 outbreak through the mediator effect of emotional dysregulation.

## 3. Methods

### 3.1. Participants and Procedures

We enrolled in the study outpatients with a documented or clinically established diagnosis of FMD, according to Gupta & Lang criteria [15], regularly followed up in the outpatients Movement Disorder Unit of Fondazione Policlinico Universitario A. Gemelli- IRCCS in Rome. We further confirmed the diagnosis according to DSM-5 criteria for Functional Neurological Disorders [17].

In addition to a strict diagnosis of FMD, inclusion criteria were as follows: (a) age 18–65 years; (b) at least 5 years of education; (c) Italian language native speaker.

Exclusion criteria were: (a) diagnosis of substance dependence or abuse in the 2 years before the assessment; (b) traumatic brain injury with loss of consciousness; (c) major medical or neurological conditions; (d) Mini-Mental State Examination (MMSE) score lower than 24 [18]; (e) any potential brain abnormality on an MRI; (f) any potential abnormality on neurophysiological tests (EEG or evoked potential test). Patients were matched one-to-two on demographic characteristics with healthy controls (HC) from the same geographical area. Exclusion criteria were the same as those for the patient group.

The study was reviewed and approved by the local ethics committee. All participants provided informed written consent.

### 3.2. Data Collection

In this sample, we used a semi-structured interview to get information on COVID-19 symptoms and to assess the impact of the COVID-19 pandemic on the disease perception. A senior psychiatrist, together with a senior neurologist, carried out the semi-structured interview. The final assessment was based on information from caregivers and from any medical documentation, too. The interview consisted of: (a) socio-demographic and clinical data. In particular, we collected the age at which the individual first experiences psychiatric symptoms, disease duration, the phenomenology of the FMD, neuropsychiatric current pharmacological treatment, psychotherapy, physical therapy, botulinum toxin therapy and disability measures; (b) COVID-19-related questions, mainly focused on its effect on the disease burden, in particular on neurological symptoms, emergency neurological assistance, pharmacological treatment break-off or physiotherapy withdrawal. All data collected were entered in preprinted medical records.

### 3.3. Psychometric Assessment

K10. We adopted the Kessler Psychological Distress Scale (K10) [19] in order to evaluate psychological distress in our sample. K10 is a 10-item questionnaire assessing the general distress experienced by participants in the last month. The Italian version of the scale was utilized [20]. Low and high levels of psychological distress are reported by low and high scores, respectively.

DERS. We used the Difficulties in Emotion Regulation Scale, brief version (DERS-18) [21], to self-report and assess emotion dysregulation levels. Items refer to difficulties in accepting emotional responses, difficulties in goal-directed behaviors, lack of impulse and emotional control, difficulties in regulating emotions and lack of emotional clarity. Items were assessed according to the validated Italian version [22]. The summary score resulted from the sum of each item. Great difficulties in regulating emotions are reported by high DERS scores. Previous studies demonstrated that the scale has a convergence validity in assessing several outcomes associated with emotion dysregulation [21].

TEMPS-A-39. We adopted the Italian version of the 39-item Temperament Evaluation of Memphis, Pisa, Paris and San Diego (TEMPS-A-39) [23], a self-rated questionnaire composed of 39 items, investigating the individual representation of 5 affective temperaments: cyclothymic, depressive (dysthymic), irritable, hyperthymic and anxious temperament. The score on each temperament is obtained by summing Yes responses. The 39-item version has shown a five-factor solution as the best fit [8].

### 3.4. Statistical Analysis

We compared individuals with FMDs and HC on demographic and COVID-19-related information on the basis of the chi-square test for nominal variables and one-way analysis of variance (ANOVA) for continuous variables. Then, we performed a multivariate analysis of variance (MANOVA), with psychological distress and psychopathological characteristics as dependent variables and the diagnostics groups as independent factors. We performed a sequence of ANOVAs in order to test differences between the considered groups on dependent variables using the same covariates. We corrected for multiple comparisons using the Bonferroni procedure (*p* < 0.05/number of comparisons) to minimize the likelihood of type I errors.

#### Mediation Analysis

In the patients group, we tested whether the correlation between the prevalent affective temperament in FMDs and psychological distress is mediated by emotional dysregulation using the PROCESS macro for SPSS for mediation analyses [24]. We tested the hypothesis that temperament did not cause psychological distress directly but through a mediator factor (emotional dysregulation). We presented this relation between the mediator, the independent factor and the dependent one using path diagrams. The regression coefficients (betas) were also presented.

We used a bootstrap model to resample the original sample and test the mediation analysis on potential biases [25]. We used 10,000 bootstrap samples to determine the 95% confidence interval of the direct and indirect, with outcomes considered significant if confidence intervals do not include 0. *p*-value was set at <0.01. All statistical analyses were performed using SPSS v. 25 (IBM Corp., Armonk, NY, USA).

## 4. Results

In total, 96 individuals (32 patients and 64 HCs) were included in this study according to inclusion/exclusion criteria. Demographic, clinical and psychopathological characteristics and COVID-19-related information are reported in Table 1.

Patients with FMD and HC were not different in demographic characteristics. In the FMD group, there was a high prevalence of female sex (84.4%); the mean age at onset was 41.68 years (SD = ±17.69), with a mean disease duration of 10.62 years (SD = ±9.52). The majority of patients presented with functional dystonia (45.4%) and tremor (27.3%), with most of them presenting at least one body district affected. In 18.2% of cases, patients showed combined FMD with two or more movement disorders.

Overall, 31.3% and 40.6% of the patients reported the need for urgent neurological care and subjective worsening of neurological symptoms during the COVID-19 pandemic, respectively. Furthermore, 28.1% of patients reported discontinuation of a previous pharmacological treatment or physiotherapy program due to the COVID-19 outbreak.

MANOVA demonstrated a significant effect (Wilks’ Lambda = 0.66, F = 5.41, df = 8, *p* < 0.001) on the psychopathological characteristics of the two groups. A series of ANOVAs highlighted that the FMD group significantly experienced more psychological distress during the COVID-19 pandemic, more emotional dysregulation and were more cyclothymic than HC (Table 1).

**Figure 1:**
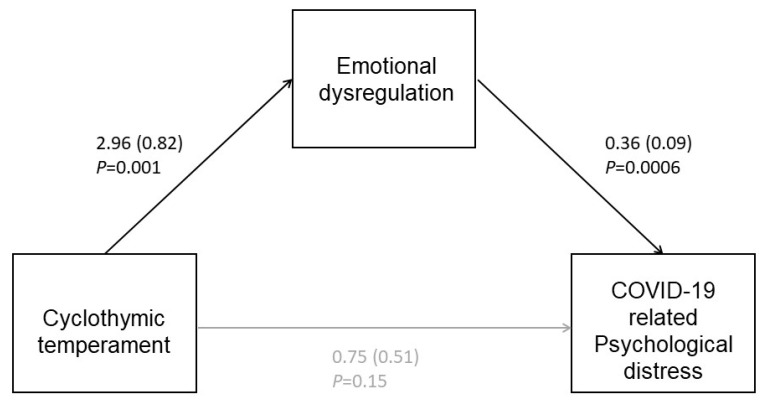
The mediation model showed the link between cyclothymic temperament and psychological distress, with the intervention of emotional dysregulation.

The mediation model showed that cyclothymic temperament was positively associated with emotional dysregulation, which in turn was positively associated with psychological distress (Figure 1). According to the bootstrapped 95% confidence interval, the significant indirect effect was 1.08 (LLCI =0.41, ULCI =2.41). Moreover, we did not detect a significant direct effect of cyclothymic temperament on pandemic psychological distress (LLCI = −0.29, ULCI = 1.79).

## 5. Discussion

Our study aimed at investigating the psychological impact of the COVID-19 pandemic on individuals with FMD. Our results support the general concern about the effect of the pandemic on people living with neuropsychiatric disorders. Previous data highlighted that the incidence of FMD had a peak during the COVID-19 pandemic. Specifically, a recent study identified an increase of 60.1% in the incidence of FMD [26]. Among young people, in particular, an impressive growth of functional tic-like behaviors has been observed [27]. Some authors referred to this phenomenon as an additional “silent epidemic” alongside the COVID-19 pandemic [28].

In our study, up to 31% and 41% of individuals with a previous diagnosis of FMD reported a need for urgent neurological care and worsening of neurological conditions during the COVID-19 outbreak, respectively. These results are similar to those observed by Delgado and colleagues, who found that 34% of patients with FMD presented with worsening symptoms during the pandemic [29]. Results are also in line with Machado and colleagues, who highlighted higher rates of hospitalization for severe cases of FMD during the pandemic [28]. Moreover, subjects with FMD displayed increased psychological distress, as compared to HC. In 2020, a study on a sample of 10 patients with FMD and eight patients with psychogenic non-epileptic seizures (PNES) showed higher levels of anxiety and stress symptoms related to COVID-19 in patients compared to HC [30]. These results are also consistent with the observation that patients with FMD are more susceptible to developing stress and anxiety symptoms than HC, particularly in stressful situations [9,31].

Conversely, a recent study found that patients with FMD might not show a specific vulnerability due to the restrictive measures imposed during the COVID-19 pandemic. In addition, when comparing periods with different severity in social restrictions, authors found no changes in the severity of symptoms. The worsening of pain also resulted as a predictor factor for the worsening of motor symptoms. Authors hypothesized that, in this particular case, patients might have been distracted by a diverted self-focused attention/monitoring toward the stressful global pandemic, which, in turn, led to the stability of motor symptoms [32].

In our sample, individuals with FMD were more cyclothymic and exhibited decreased emotion regulation abilities. Among the same group of subjects, we also found a direct association between cyclothymic temperament and psychological distress, which resulted indirect and mediated by impaired emotion regulation mechanisms.

Considering temperament as the component involved in the individual’s variability for emotional reactivity, there is evidence that differences in temperament and character may distinguish patients with FMD from others [33]. Cyclothymic temperament is operationalized by abrupt changes in mood, behavior, vitality and thoughts. Difficulties in emotion regulation may prevent cyclothymic individuals from regulating their feelings against stressful stimuli in a balanced way. This can cause individuals with a cyclothymic temperament to experience rapid and unexpected mood swings in response to stress, including the one caused by COVID-19 [34]. Consistent with this conceptual framework, the redirection of affect representation in bodily symptoms upon emotional stress has been underlined in recent models and definitions of FMD [35]. Cognitive neuroscience highlights that the sensory-motor system plays a key role in shaping emotional responses, including emotional expression and recognition [36,37]. Accordingly, compromised neuronal emotion processing in patients with FMD is suggested by an imbalance of frontocortical and sensorimotor network activity in response to emotionally salient stimuli [38]. From a neuropsychological perspective, a deficit in the theory of mind, considered as the ability to attribute mental states, was detected in FMD patients [39]. One may speculate that individuals with FMD who display impaired emotion regulation ability may perceive an increased level of stress during the COVID-19 pandemic, which, in turn, may ultimately lead to the worsening of neurological symptoms. This hypothesis is in accordance with former findings in the general population, which showed that emotional dysregulation is specifically linked with psychological distress due to the COVID-19 pandemic [7,40].

## 6. Study Limits and Conclusions

Before presenting our conclusions, we must disclose some limitations that might potentially limit the generalizability of our results. Indeed, the study involved a relatively small sample size, with self-selection bias and self-reported outcome measures. Furthermore, it lacks longitudinal follow-up. Conversely, a major strength of the study is having accurately selected a group of individuals assessed according to very strict criteria [15].

Patients with FMD, in particular those with a cyclothymic temperament, are more vulnerable to the stressful effect of the COVID-19 outbreak. Our results suggest that emotional dysregulation may represent a dimension mediating this effect and provide insight for developing intervention policies.The mental health overload of the COVID-19 pandemic, caused by social isolation, financial strain and other pandemic-related burdens, could persist over the years in FMD individuals. Prospective studies with a larger sample size are therefore needed to address long-term implications. Moreover, new innovative research techniques may be applied to further expand our observation of the burden of COVID-19 on patients with FMDs. Some recent studies employed Online Photovoice (OPV) methodology to assess the quality of life during the pandemic [41,42]. Photovoice is a method in which participants take and narrate photographs to share their experiences and perspectives. Future studies may consider using OPV to better detect the burden of the pandemic in FMDs, allowing patients to express directly their own experiences.

In conclusion, our findings may set the foundation for future studies addressing mental health needs in particularly vulnerable populations like those affected by neuropsychiatric disorders and encourage patient-tailored interventions [43,44].

## Figures and Tables

**Table 1:** Demographic, clinical and psychopathological characteristics of the sample and COVID-19-related information.

Characteristics	FMD N = 32	HC N = 64	χ^2^ or F	df	*p*
**Demographical and clinical characteristic**					
Age (years): (mean ± SD)	51.06 (16.03)	51.04 (15.73)	0.00	1	0.99
Males: *n* (%)	5 (15.6)	10 (15.6)	0.00	1	1
Age at onset: (mean ± SD)	41.68 (17.69)	-	-	-	-
Duration of illness: (mean ± SD)	10.62 (9.52)	-	-	-	-
Neuropsychiatric pharmacological treatment: *n* (%)	18 (56.3)	-	-	-	-
Psychotherapy: *n* (%)	9 (28.1)	-	-	-	-
Physical therapy: *n* (%)	20 (62.5)	-	-	-	-
Botulinum toxin therapy: *n* (%)	10 (31.3)	-	-	-	-
IADL: (mean ± SD)	6.06 (2.56)	-	-	-	-
**COVID-19 related information**					
Subjective worsening of neurological symptoms during COVID-19 pandemic: *n* (%)	13 (40.6)	-	-	-	-
Need of urgent neurological care during COVID-19 pandemic: *n* (%)	10 (31.3)	-	-	-	-
Pharmacological treatment break-off or physiotherapy withdrawal during COVID-19: *n* (%)	9 (28.1)	-	-	-	-
**Psychopathological characteristics**					
Psychological distress (K-10) during COVID-19 pandemic: (mean ± SD)	27.75 (10.34)	18.81 (5.53)	30.15	1	<0.001
Emotional dysregulation (DERS): (mean ± SD)	42.09 (16.55)	31.25 (10.09)	15.80	1	<0.001
TEMPS-A Cyclothymic: (mean ± SD)	5.18 (3.06)	2.82 (2.70)	14.84	1	<0.001
TEMPS-A Depressive: (mean ± SD)	2.28 (1.83)	1.93 (2.21)	0.57	1	0.45
TEMPS-A Irritable: (mean ± SD)	1.12 (1.36)	1.00 (1.28)	0.19	1	0.66
TEMPS-A Hyperthymic: (mean ± SD)	4.28 (1.70)	3.96 (1.84)	0.64	1	0.42
TEMPS-A Anxious: (mean ± SD)	1.18 (1.22)	1.39 (1.13)	0.64	1	0.42

Legend: Significant results in **bold** (after Bonferroni correction for multiple comparisons). SD, standard deviation; df, degrees of freedom; χ^2^,chi squared test; *p*, statistical significance; F, value of variance of the group means; IADL, Instrumental activities of daily living; K-10, Kessler Psychological Distress Scale; DERS, Difficulties in Emotion Regulation Scale TEMPS-A, Temperament Evaluation of Memphis, Pisa, Paris and San Diego Autoquestionnaire.

## Data Availability

Anonymized data of this study could be available on reasonable request after approval of their requests.
